# Scaling trajectories of cities

**DOI:** 10.1073/pnas.1906258116

**Published:** 2019-06-24

**Authors:** Marc Keuschnigg

**Affiliations:** ^a^The Institute for Analytical Sociology, Department of Management and Engineering, Linköping University, SE-601 74 Norrköping, Sweden

**Keywords:** dynamics of cities, spatial inequality, urban scaling, science of cities

## Abstract

Urban scaling research finds that agglomeration effects—the higher-than-expected outputs of larger cities—follow robust “superlinear” scaling relations in cross-sectional data. But the paradigm has predictive ambitions involving the dynamic scaling of individual cities over many time points and expects parallel superlinear growth trajectories as cities’ populations grow. This prediction has not yet been rigorously tested. I use geocoded microdata to approximate the city-size effect on per capita wage in 73 Swedish labor market areas for 1990–2012. The data support a superlinear scaling regime for all Swedish agglomerations. Echoing the rich-get-richer process on the system level, however, trajectories of superlinear growth are highly robust only for cities assuming dominant positions in the urban hierarchy.

Urban scaling has evolved into an important paradigm for the study of socioeconomic agglomeration effects (1–3). It finds urban outputs to possess robust scaling relations with population size and captures inequalities between cities with a power-law function $Y\left(N\right)∼Y_{0}N^{\beta }$, where Y is a socioeconomic quantity’s city-wide total, $Y_{0}$ a baseline common to all cities, N city size, and β a multiplier indicating the percentage change in Y following a 1% increase in N. Superlinear scaling (β>1) has been found in urban systems on different continents (1, 2) based on cross-sectional data comparing cities of different sizes at a given point in time. Still, the paradigm has predictive ambitions involving the scaling trajectories of individual cities over time, presuming urban attributes to change as cities gain in population and treating cities that at time t have very different sizes as self-similar “scaled versions of one another” (1), expected to go through similar growth trajectories—only in different historical epochs. This theorizing implies strong connections between cross-sectional urban scaling on the system level and longitudinal scaling on the level of individual cities (4).

A dynamic approach to urban scaling has been recently pioneered based on traffic data capturing time delays in 101 US metropolitan areas over time (5). While this research is inspiring, I argue that the previously used data are inadequate for a valid test of longitudinal urban scaling. Changes in local transportation policies and evolving commuting patterns readily affect urban mobility and it is difficult to partial out local and system-wide distortions of scaling relations. This led to premature conclusions (ref. 5 reports concave scaling regimes and strong historical inertia) and provided no evidence for a single exponent governing the growth trajectories of cities.

Here, I use geocoded microdata on wage income from Swedish population registers for 1990–2012 to monitor the scaling trajectories of cities as their populations grow. My report provides compelling data to resolve this controversy and, taking a microlevel approach, provides a conceptual advance in the study of cities’ growth trajectories.

## Results

A longitudinal perspective conflates variations in city sizes with economic development and social change and, to isolate the effect of city-size variations, we must partial out concomitant socioeconomic trends. Most importantly for wages as the observed urban output, these trends include gains in gross domestic product (GDP), educational expansion, increases in female labor force participation, and changing migration patterns. To exclude a large portion of socioeconomic change, I restrict my analysis to the Swedish-born working-age male population, scrutinizing a total of 1.12 million fully employed men nested in 73 labor market areas (LMAs), Sweden’s functional demarcation of metropolitan areas (6).

**Fig. 1.**
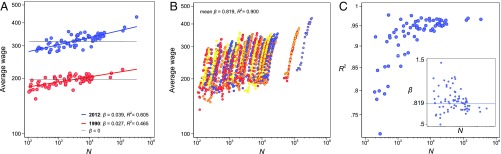
Scaling relations of per capita wage (measured in thousands of inflation-adjusted Swedish kronor) and cities’ male labor force (N). (*A*) Cross-sectional scaling for 73 Swedish LMAs in 1990 (red: β=0.027±0.007 [95% confidence interval], $R^{2}=0.465$) and 2012 (blue: β=0.039±0.008, $R^{2}=0.605$). Gray lines indicate proportional relations (β=0); the colored lines show estimates of β from a linearized model (Eq. **1** in *Materials and Methods*). (*B*) Scaling trajectories of individual LMAs. The average longitudinal β is 0.819±0.032 ($R^{2}=0.900$; Eq. **2**). (*C*) Model fit ($R^{2}$) of 73 individual regressions is highest for big cities and decreases for LMAs with fewer than 10,000 male workers. *C*, *Inset* plots LMA-specific β against population sizes and the horizontal line indicates the average scaling parameter β=0.819. For the 3 biggest LMAs, Stockholm, Gothenburg, and Malmö, longitudinal β varies between 0.695±0.070 and 0.760±0.097.

Fig. 1*A* reiterates a cross-sectional analysis for 1990 and 2012, comparing the average wage between LMAs (Eq. **1** in *Materials and Methods*; note that for per-capita outputs β>0 signifies superlinearity). In 1990, the scaling relation amounts to β=0.027±0.007 and population size explains 47% of wage differences between LMAs. Doubling a city’s male labor force N in 2012 relates to a 3.9% ±0.8 increase in average wage ($R^{2}=0.605$). Superlinearity increases substantially during the 23-y period. Important factors for the surge in spatial inequality are the outmigration of talented people from small towns in Sweden, crucially adding productivity to the largest cities (6), and the growing concentration of specialist service industries, with high value added per worker, in cities atop the urban hierarchy (7).

Fig. 1*B* displays the scaling trajectories of individual cities. The size of the male labor force increased steadily in all labor market areas such that each LMA’s N scale translates roughly into a 23-y timescale. The trajectories are approximately linear and my estimate of the average longitudinal β is 0.819±0.032 ($R^{2}=0.900$). I find a superlinear scaling regime for all LMAs but model fit is higher for larger cities (Fig. 1*C*). Superlinear growth is less robust in smaller places and Fig. 1*C*, *Inset* plots the variation of estimated β against population sizes. For the 3 biggest LMAs, Stockholm, Gothenburg, and Malmö, β varies between 0.695±0.070 and 0.760±0.097. For places with N < 10,000 fully employed male workers (corresponding to a full population of approximately 75,000) variation in β increases. These differences are not due to variations in sample size.*

So far, the trajectories include wealth creation due to economic development and social change. Inter alia, Sweden experienced 2 economic downturns in 1990–1993 and 2008–2012, leaving visible imprints—slight S curves—on cities’ growth trajectories. Table 1 presents a stepwise approximation of the net wage-size relation. The slope of the longitudinal scaling decreases under statistical control for important aspects of socioeconomic change: Model 2 is based on aggregate city data (Eq. **2**) and partials out system-wide changes in GDP per capita and educational expansion, reducing β to 0.191±0.047. The underlying microdata permit more granular statistical control, including differences in the composition of local labor forces and the productivity-related changes that workers experience over time. Model 3, based on 16.8 million data points tracing the earning paths of 1.12 million employees (Eq. **3**), reproduces the scaling parameter from the aggregate analysis. Model 4 further controls for microlevel measures of workers’ investments in human capital, increases in work experience, occupational changes from public to private sector employment, unemployment, and migration between LMAs, reducing β to 0.094±0.002. This estimate holds for both smaller (N< 10,000: β=0.101±0.002) and larger LMAs (N ≥ 10,000: β=0.095±0.002).

**Table 1.** Estimates of longitudinal urban scaling decrease under control for economic development and social change

	Aggregate data		Microdata	
Independent variables	1	2	3	4
log(N)	0.819	0.191	0.207	0.094
GDP per capita		0.018	0.043	0.013
Mean education		0.210		
Education				0.222
Experience				0.063
${Experience}^{2}$				−0.002
Employed				0.754
Private sector job				0.165
Migration between LMAs				0.037
$R^{2}$ within	0.900	0.963	0.168	0.308

Dependent variable: log(wage). Shown are longitudinal regressions on aggregate data with 73 × *T* = 1, 679 city years (models 1 and 2, based on Eq. **2**) and on microdata with 1.12 million × ${\overset{-}{T}}_{i}$ = 16.8 million person years (models 3 and 4, based on Eq. **3**). The coefficients for log(*N*) indicate the mean *β* within the 73 LMAs. Model 4 yields the closest approximation of the true longitudinal wage-size scaling relation.

## Discussion

By design, the full microdata model approximates most closely the established cross-sectional interpretation of β: Doubling population size, ceteris paribus, increases average wage by 9.4% ±0.2. The estimate of longitudinal scaling is much larger than its cross-sectional counterpart (3.9% ±0.8). Hence, moving forward in time within a given city correlates with greater increases in wealth creation than does moving in space between cities. The imbalance between cross-sectional and longitudinal scaling casts doubt on the scale invariance of urban growth, supporting the notion that cities’ temporal dynamics differ from the spatial dynamics of a city system.

In favor of the paradigm’s predictive ambitions I find that superlinear scaling governs the trajectories of all agglomerations in Sweden’s urban system. At the same time, I also find superlinear growth to be nonrobust in LMAs with fewer than 75,000 inhabitants and the power law’s empirical fit much better for larger cities than for smaller ones. This qualitative difference signifies cities’ various positions in an urban hierarchy (4, 7, 8) and the disparities in industrial structures, sociodemographic composition, and migration flows (6, 9, 10) separating smaller from larger agglomerations. Only when such disparities are controlled for (Table 1, model 4), longitudinal scaling parameters are almost identical for small and for large cities. My findings suggest that dominant positions in the urban hierarchy give an advantage to larger cities and that this path dependency places bounds on the self-similarity of growth trajectories within an urban system.

## Materials and Methods

### Data.

I use geocoded microdata assembled by Statistics Sweden covering the country’s entire urban system represented by 75 LMAs that, from the smallest (2,673 inhabitants) to the largest (2.51 million inhabitants), span 4 orders of magnitude. Government agencies, including tax authorities and educational institutions, collected and directly reported the data.

I exclude all workers from the mining areas Gällivare and Kiruna in the far north of Sweden, whose wages depend primarily on the presence of natural resources. I further restrict my analysis to the 1.12 million Swedish-born males aged 18–60 y fully employed for at least 2 y during 1990–2012. I exclude women because of both marked fluctuations in female labor force participation (the maximum percentage point difference is 21.1 for women, but only 6.7 for men) and a shrinking gender wage gap (the unconditional gender wage gap decreased from 34% in 1990 to 25% in 2012). I also exclude foreign-born men because of a strong increase in the migrant population in Sweden (from 9.2% of the full population in 1990 to 15.4% in 2012) and the concomitant changes in immigrants’ labor force participation and the varying disadvantages they face along different career paths. These system-wide trends must not affect the longitudinal estimation of β, and so my restrictions control for a large portion of socioeconomic change during the period of observation. N thus represents the size of the male labor force in each LMA at time t. During the 23-y period the sample population rose from 459,338 to 1,125,677 (a 2.45-fold increase) and, between LMAs, the ratio N (2012)/N (1990) varies from 1.65 to 2.85.

I use individuals’ gross annual wage income (in thousands of Swedish kronor, inflation adjusted with base year 2012) as an indicator of local wealth creation. Average annual wage increased 1.66-fold, from 195,000 kronor in 1990 to 324,000 kronor in 2012. In the city-level analysis (Eqs. **1** and **2**) I aggregate residents’ wages into their respective city’s average wage.

### Models.

To estimate cross-sectional β (Fig. 1*A*), I linearize $Y_{j}\left(N\right)∼Y_{0}N_{j}^{\beta }$ and reformulate the power law on the per-capita level:

$$\log\left(\frac{Y_{j}}{N_{j}}\right)=\log\left(Y_{0}\right)+\beta \log\left(N_{j}\right)+\epsilon_{j}.$$ [1]

The dependent variable is now the logarithm of an average attribute of LMA j=1,2,…,M (M=73), and $\epsilon_{j}$ is a normally distributed error with zero mean capturing each city’s distance to the predicted power-law function. Note that the transformation to a per-capita measure of urban output changes the threshold for superlinear scaling to β>0.

To trace the scaling trajectories of M individual cities (Fig. 1*B*), I substitute t for j and—taking all 23 data points for each city separately—estimate Eq. **1** for each j over time t=1,2,…,T (T=23). To derive the average longitudinal β, I can combine those estimations using a single longitudinal regression with an additional error term $\alpha_{j}$ on the city level (11), capturing each city’s mean deviation from the common baseline $\log\left(Y_{0}\right)$ and—by giving each city its own intercept—absorbing all time-constant factors that affect a city’s average income (e.g., geographic location and historical inertia):

$$\log\left(\frac{Y_{jt}}{N_{jt}}\right)=\alpha_{j}+\beta \log\left(N_{jt}\right)+\epsilon_{jt}.$$ [2]

This longitudinal β indicates the average wage-size scaling relation of single cities over time. Technically, the parameter is estimated after demeaning each city’s trajectory (eliminating between-city variance) and β is determined exclusively from within-city variance over time.

The microlevel version of Eq. **2** is based on 16.8 million data points tracing the earning paths of 1.12 million employees during 1990–2012 and predicts individual wage $y_{i}$ conditional on $N_{i}$ at time t:

$$\log\left(y_{it}\right)=\alpha_{i}+\beta \log\left(N_{it}\right)+\epsilon_{it}.$$ [3]

The unit-specific intercept $\alpha_{i}$, now located on the individual level, absorbs employees’ time-constant characteristics (e.g., cognitive ability, family background) and β captures the average effect that changes in $\log\left(N_{it}\right)$ have on $\log\left(y_{it}\right)$ based on variance within each individual’s trajectory.

These models permit the adding of control variables to partial out socioeconomic change and to approximate the net size effect on per capita wage. To the aggregate-level model (Eq. **2**), I add GDP per capita (in thousands of constant 2011 international dollars) and educational expansion (the local population’s average years of education) with values for each city at t. To the individual-level model (Eq. **3**), I add more granular control variables with values for each employee at t, including educational attainment and work experience (measured as additional years during the observation period) as well as binary measures of employment status (0 = unemployed, 1 = employed), employer type (0 = public, 1 = private), and residential moves. The latter indicator (0 for all person years in the native LMA, 1 for all person years in another LMA) absorbs variations in an individual’s assigned city size $N_{it}$ due to migration between LMAs. All control variables carry the expected coefficients (Table 1): On the city level, GDP per capita and average educational levels correlate positively with aggregated per capita wage. On the individual level, each additional year of education associates with 22.2% higher wages on average and—also in line with the human capital earnings function (12)—work experience (up to approximately 16 y) associates with increased pay. Being fully employed (vs. unemployed) raises individual wages by 75.4% and private-sector (vs. public sector) employment by 16.5%, on average. Migration between LMAs results in 3.7% higher wages on average for all years after leaving the native LMA. All estimates are significant at P< 0.001 (using cluster-robust standard errors).

## Footnotes

*Computing each LMA’s average wage from a random sample of only 100 workers yields an average longitudinal β of 0.817±0.034; among the 3 biggest LMAs β varies between 0.687±0.098 and 0.829±0.110, and the patterns from Fig. 1*C* remain unchanged.
